# Biomarkers That Seem to Have the Greatest Impact on Promoting the Formation of Atherosclerotic Plaque in Current Scientific Research

**DOI:** 10.3390/cimb46090564

**Published:** 2024-08-29

**Authors:** Maksymilian Kłosowicz, Dawid Leksa, Dorota Bartusik-Aebisher, Angelika Myśliwiec, Klaudia Dynarowicz, David Aebisher

**Affiliations:** 1 English Division Science Club, Medical College, University of Rzeszów, 35-310 Rzeszów, Poland; klosowiczmaksymilian@gmail.com; 2 Department of Photomedicine and Physical Chemistry, Medical College, University of Rzeszów, 35-310 Rzeszów, Poland; 3 Rzeszów Center for Vascular and Endovascular Surgery, 35-010 Rzeszów, Poland; dleksa@gmail.com; 4 Department of Biochemistry and General Chemistry, Medical College, University of Rzeszów, 35-310 Rzeszów, Poland; 5 Center for Innovative Research in Medical and Natural Sciences, Medical College, University of Rzeszów, 35-310 Rzeszów, Poland; amysliwiec@ur.edu.pl (A.M.); kdynarowicz@ur.edu.pl (K.D.)

**Keywords:** atherosclerosis, circulatory system, biomarkers

## Abstract

Atherosclerosis is a chronic inflammatory disease that causes degenerative and productive changes in the arteries. The resulting atherosclerotic plaques restrict the vessel lumen, causing blood flow disturbances. Plaques are formed mainly in large- and medium-sized arteries, usually at bends and forks where there is turbulence in blood flow. Depending on their location, they can lead to various disease states such as myocardial infarction, stroke, renal failure, peripheral vascular diseases, or sudden cardiac death. In this work, we reviewed the literature on the early detection of atherosclerosis markers in the application of photodynamic therapy to atherosclerosis-related diseases. Herein, we described the roles of C-reactive protein, insulin, osteopontin, osteoprotegerin, copeptin, the TGF-β cytokine family, and the amino acid homocysteine. Also, we discuss the role of microelements such as iron, copper, zinc, and Vitamin D in promoting the formation of atherosclerotic plaque. Dysregulation of the administered compounds is associated with an increased risk of atherosclerosis. Additionally, taking into account the pathophysiology of atherosclerotic plaque formation, we believe that maintaining homeostasis in the range of biomarkers mentioned in this article is crucial for slowing down the process of atherosclerotic plaque development and the stability of plaque that is already formed.

## 1. Introduction

The most serious risk factor for atherosclerosis and other cardiovascular diseases is age [1,2,3]. Cardiovascular diseases are the cause of death in over 40% of patients in the age group > 65 years [4,5,6,7]. Other risk factors for the development of atherosclerosis include obesity, diabetes, hypertension, cigarette smoking, low physical activity, positive family history, chronic kidney disease, dyslipidemia with increased levels of LDL-C, Lp(a) and triglycerides and decreased HDL-C, chronic inflammation, and psychological and social factors [8,9]. Gender also plays a role in the development of atherosclerosis. Joshua J. Man et al. report that in men, atherosclerotic plaques appear faster, and are composed of a larger number of inflammatory cells and therefore more unstable than in women, which contributes to a greater risk of cardiovascular events [10].

## 2. Mechanism of Atherosclerotic Plaque Formation

**Figure 1:**
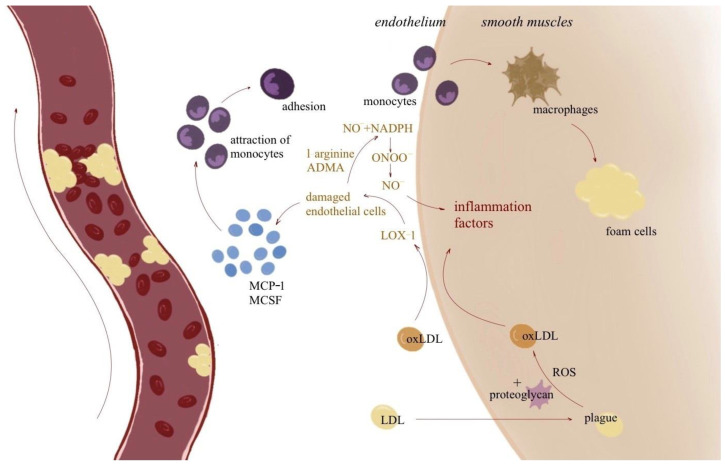
The mechanism of plague formation. Abbreviations: Monocyte Chemoattractant Protein-1 (MCP-1); macrophage colony-stimulating factor (MCSF); reactive oxygen species (ROS); LOX-1, the receptor for oxidized low-density lipoprotein.

Atherosclerotic plaque consists of lipid molecules, inflammatory cells, smooth muscle cells, and extracellular matrix (Figure 1). These substances accumulate in the inner lining of the arteries [4].

A special role in this process is played by vascular endothelial cells (ECs), which ensure the appropriate tension of the vessel wall, aid smooth muscle proliferation, regulate the permeability of compounds between blood and tissues, and have an antithrombotic function [11]. Disturbed blood flow has the effect of promoting the formation of atherosclerotic plaques. This contributes to the increased proliferation of endothelial cells, reduced migration, increased apoptosis, accelerated aging, and impaired repair in arteries. Disturbed flow causes increased expression of the adhesion molecules VCAM1, ICAM1, and E-selectin, increased activation of the NF-κB signaling pathway, and increased production of the pro-inflammatory cytokines IL-1, IL-6, and CCL5, which leads to further intensification of inflammation. Finally, endothelial cells change their spatial arrangement and shape to adapt to the turbulent blood flow. The cells lose their uniform arrangement and take on large shapes. These changes are accompanied by the restructuring of the entire cytoskeleton and the arrangement of cellular organelles within the cell [12]. Damage to the vascular wall with disruption of endothelial cell continuity results in the deposition of oxidized low-density lipoprotein (LDL-ox) deposits, which then increase the production of chemokines and pro-inflammatory cytokines, which cause the influx of additional inflammatory cells into the damaged wall [11,13].

Among the inflammatory cells, monocytes play an extremely important role, as they are among the first to migrate to the site of a damaged vessel, transforming into macrophages. Then, they absorb ox-LDL particles accumulated in the inner layer of the artery and undergo a transformation into foam cells, which are the main building blocks at the early stage of atherosclerotic plaque formation [1]. Incoming macrophages can be divided into two main groups. M1 macrophages secrete a number of pro-inflammatory cytokines, such as NF-kB and AP IL-1, IL-6, IL-8, IL-12, IL-18, and TNF, and reactive oxygen species (ROS), which intensify inflammation. M2 macrophages reduce inflammation by producing anti-inflammatory cytokines such as IL-4, IL-13, fibronectin, and TGF-β. The process of differentiating incoming macrophages into specific subtypes is caused by polarization induced by LDL-ox. The mutual ratio and distribution of M1 and M2 macrophages determines the stability of the atherosclerotic plaque. The M2 subtype predominates in stable plaques, while the M1 subtype predominates in those at risk of rupture more frequently. Inflammation and the presence of LDL-ox induce the formation of foam cells [5,14,15].

The presence of SRA1, CD36, and LOX1 receptors is crucial for the formation of foam cells, thanks to which LDL-ox particles can be absorbed by macrophages. Within the lysosomes, LDL-ox is converted to free cholesterol and fatty acids, which are then transported to the endoplasmic reticulum where they undergo the process of re-esterification [14].

In the case of atherosclerosis, the phenotype of the smooth muscle cells (SMCs) of the vascular wall also changes. The change in phenotype leads to disturbances in the differentiation, proliferation and migration of individual cells. Huize Pan et al., in their study, suggested that the change in the phenotype of SMC cells to SMC-derived cells (SDCs) resembles malignant transformation. There is instability within the genome, which triggers the processes of uncontrolled division and stopping cell death. It has also been shown that a deficiency of the TRP53 protein, which is one of the main regulators of cell death and controls cell division, results in a reduction in apoptosis and, consequently, an increase in atherosclerotic plaque [16,17].

Another extremely important component in the process of atherosclerotic plaque formation is the role of smooth muscle cells in the vascular wall. Physiologically, smooth muscles are responsible for regulating blood pressure and the appropriate tension of the vascular wall. In the case of atherosclerosis, they participate in the stabilization of the atherosclerotic plaque [18,19,20]. Based on their structure, atherosclerotic plaques can be divided into three types. The first one is characterized by a thick fibrous cap with the presence of numerous SMCs, a small amount of inflammatory infiltrate, and a large lipid–necrotic core. The second group is the so-called unstable atherosclerotic plaque, which is characterized by a thin fibrous cover that is infiltrated by numerous inflammatory cells, similar to a necrotic core. The third type is plaque susceptible to rupture. It is composed of numerous inflammatory cells, macrophages, and T lymphocytes [13].

## 3. Selected Biomarkers of Atherosclerosis

The method of detecting two or more biomarkers to prediction the development of the formation of atherosclerotic plaque is more detailed than using each of these markers individually; it shortens the patient’s hospital stay and increases the percentage of patients correctly discharged from hospital. It should also be mentioned that a more detailed assessment of the risk of developing the disease using two markers is not characterized by an increased clinical danger for the patient compared to the determination of a single marker.

### 3.1. Proteins, Peptides, and Amino Acids

Herein, we describe the role of C-reactive protein, insulin, osteopontin, osteoprotegerin, copeptin, the TGF-β cytokine family, and the amino acid homocysteine.

#### 3.1.1. C-Reactive Protein

C-reactive protein (CRP) is an acute phase protein whose level increases during inflammatory diseases. Factors influencing the level of CRP in the blood include age, gender, body weight, lipid metabolism, blood pressure, and smoking. CRP is currently used as a marker and early prognostic factor in cardiovascular disease and stroke. C-reactive protein is involved in, among others, the pathogenesis of congestive heart failure, atrial fibrillation, aortic valve diseases, and myocarditis, and the development of atherosclerotic plaques [21,22].

The effect of C-reactive protein on atherosclerotic plaques is not clear. On the one hand, CRP can induce the apoptosis process by influencing the production of pro-apoptotic cytokines such as interleukin-1β, interleukin 6, interleukin 8, tumor necrosis factor, or active oxygen radicals. It also leads to the upregulation of the p-53 protein in monocytes, which also has apoptotic properties. It further causes reduced nitric oxide production by reducing eNOS activity [23]. All these effects appear to be associated with adverse factors that increase cardiovascular risk. A study conducted by Dewa Ayu Swastini et al. showed that there is a relationship between CRP and the degree of advancement of atherosclerosis and its more widespread course. Subsequent studies have shown that CRP is a strong predictor of cardiovascular risk. Research results suggest that CRP levels are positively correlated with triglycerides and high BMI [24].

On the other hand, many scientific studies show an atheroprotective effect of CRP on the formation of atherosclerotic plaques. CRP was found together with LDL particles and macrophages within atherosclerotic plaques in people with atherosclerosis. Studies have shown that CRP molecules are able to bind oxidized LDL particles. This reaction is possible due to the presence of phosphocholine-binding sites on the CRP molecule. CRP has five such sites through which it can bind to various molecules. The impact of this combination on the formation of atherosclerotic plaque is not clear. It is currently suggested that the CRP-ox-LDL combination causes reduced uptake of LDL particles by macrophages and, consequently, the formation of foam cells constituting the mass of atherosclerotic plaque. It has also been shown that the production of inflammatory cytokines by macrophages is reduced when the cells are treated with a combination of CRP and ox-LDL [10]. However, the mere presence of the mentioned connection does not seem to be sufficient to stop the progression of atherosclerotic lesions. In recent studies on mice, greater attention has been paid to the protective effect on the formation of atherosclerotic plaques. It is believed that the ability of CRP to form connections with ox-LDL may become a key point in slowing down the formation of foam cells and, consequently, slowing down the process of atherosclerosis [25].

Research conducted showed that in a group of mice that received mutant CRP for 7 weeks, the development of atherosclerotic lesions was significantly slower and the total size of plaques was almost 40% smaller, compared to the control group that did not receive CRP [26].

#### 3.1.2. Osteopontin

Osteopontin is a phosphoprotein produced in the body by many different cells—osteoclasts, chondrocytes, macrophages, lymphocytes, epithelial cells, smooth muscles of the vascular wall, and others [27]. It performs many important functions; it is involved in the body’s inflammatory response, in wound healing, and in the pathophysiology of a number of cardiovascular diseases, autoimmune diseases such as multiple sclerosis and lupus, cancer, and liver and kidney diseases [28,29,30].

It has been shown that the increased expression of osteopontin is associated with the formation and greater instability of atherosclerotic plaques. This effect is dependent on the increased influx of macrophages and T lymphocytes within the developing lesions and their increased proliferation. Osteopontin is also responsible for reducing NO synthetase activity, contributing to reduced nitric oxide production. It also participates in the regulation of inflammation by reducing the expression of interleukin 10, which is an anti-inflammatory cytokine [31]. Additionally, it affects the Rho/Rho kinase pathway within the smooth muscles of the vascular wall, which is responsible for many important functions such as cell proliferation, regulation of contractility, and adhesion with other cells [32].

Additionally, osteopontin also contributes to increased proliferation within the intima of the blood vessel immediately after vessel revascularization. This is associated with the rapid growth of atherosclerotic plaque and the phenomenon of restenosis after cardiac intervention [33].

Increased osteopontin concentration has been demonstrated in diseases that are closely correlated with the development of atherosclerosis—arterial hypertension, prediabetes and diabetes, obesity, and chronic kidney disease. It has been shown that increased osteopontin concentration may be an independent risk factor for cardiovascular diseases [34].

The research conducted by [Xu Huang et al.] showed increased expression of osteopontin in the smooth muscles of the vascular wall, and therefore it was concluded that this may be the next therapeutic target in the treatment of atherosclerosis. For example, it has been shown that the activation of peroxisome proliferator-activated receptor δ (PPARδ) can effectively stop both the migration of muscle cells and their increased apoptosis under the influence of ox-LDL particles [35]. This mechanism can be used as part of anti-atherosclerotic treatment [36].

#### 3.1.3. Osteoprotegerin

Osteoprotegerin (OPG) in the human body is produced by various types of cells and tissues—cardiomyocytes, vascular wall cells, lungs, bones, cells of the immune system, and kidneys [37]. OPG, by binding to the receptor of nuclear factor-kB ligand (RANKL), is involved in the regulation of the proliferation and differentiation of osteoclasts derived from hematopoietic stem cells [38]. OPG, RANKL, and receptor activator of nuclear κB (RANK) are involved in bone tissue remodeling. While RANKL stimulates osteoclast maturation and activity, OPG is designed to inhibit this process by binding to RANKL [39].

Elevated OPG levels are observed in many disease states such as rheumatoid arthritis, Crohn’s disease, Kawasaki disease, and lupus [40]. The involvement of the OPG-RANKL-RANK axis may also be involved in the pathogenesis of metabolic diseases such as type II diabetes and its associated insulin resistance and metabolic syndrome, as well as non-alcoholic fatty liver disease (NAFLD) [41,42]. Studies conducted with denosumab—a monoclonal antibody aimed at reducing RANKL activity—showed that after a year of drug therapy, patients noticed a decrease in the level of glycated hemoglobin (HbA1c) and a decrease in the Homeostatic Model Assessment (HOMA-IR) index [43]. Another study also showed that denosumab is effective in patients with prediabetes, leading to a reduction in blood glucose levels and reduced insulin resistance [44].

Recent studies have shown that the OPG-RANKL-RANK axis plays a significant role in the development of atherosclerosis and calcification of blood vessels [45,46].

Subsequent studies have positively correlated OPG, RANK, and RANKL levels with acute myocardial infarction, ischemic stroke, and myocardial failure [47]. Plasma OPG levels have been shown to be an independent factor in 10-year cardiovascular risk and mortality. The results of the above studies also suggest that OPG could be used as a predictor of the development of heart failure [46].

Straface G et al. showed that the levels of plasma osteoprotegerin were higher in people suffering from atherosclerosis. Going further, they also showed that these levels were higher in people with unstable atherosclerotic plaques compared to people with stable lesions [47]. OPG levels have been further correlated with left ventricular muscle hypertrophy [48].

The pro-atherosclerotic effect of OPG is related not only, as mentioned above, to a greater cardiovascular risk and the development of diabetes or hypertension, but also to its direct molecular mechanism. Increased OPG levels are associated with increased endothelial dysfunction, leading to the apoptosis of endothelial cells and smooth muscle cells [49]. Further, OPG can block the receptor for TNF-related apoptosis-inducing ligand (TRAIL), leading to decreased NO secretion and increased release of oxygen free radicals via TRAIL [50]. Additionally, OPG interacts with vascular endothelial cells, promoting the adhesion of leukocytes and other inflammatory factors [49]. It is also suggested that OPG sensitizes vascular endothelial cells to TNF-α, which increases the migration of inflammatory cells to the intima of the vascular wall [47]. The reduced stability of atherosclerotic plaques in patients with high OPG levels is associated with the excessive production of metalloproteinases and a general pro-inflammatory effect [50]. It has also been shown that the level of OPG is correlated with increased vessel stiffness due to their excessive calcification [46]. Despite the above reports, the exact mechanism of the promoting effect of OPG on the development of atherosclerotic plaques is still poorly understood [51].

#### 3.1.4. Insulin Resistance

Insulin is believed to have a protective effect against the development of atherosclerosis. It increases the availability of nitric oxide, which affects the functioning of the vascular endothelium, but too high concentrations may cause the opposite effect. Insulin resistance is correlated with the increased production of free oxygen radicals and increased expression of adhesion molecules on endothelial cells. All these changes are involved in the early stages of atherosclerotic plaque formation. The insulin resistance that develops in the body also leads to an increased inflow of leukocytes into the walls of blood vessels [52,53]. Mitochondrial dysfunction and, consequently, increased oxidative stress, are other mechanisms by which high insulin levels resulting from insulin resistance promote the formation of atherosclerotic plaques. Excess insulin further leads to increased lipogenesis, fat accumulation, and its increased deposition in artery walls. By increasing peptides such as angiopoietin 2 and endothelin-1, inflammation in blood vessels increases and hypertension develops, respectively [54].

Hyperinsulinemia and its accompanying hyperglycemia are associated with the increased release of vasoconstrictive substances and inflammatory factors promoting vascular endothelial dysfunction. This effect is also realized through lipotoxicity, which is demonstrated by high levels of insulin. The presence of an increased level of free fatty acids not only directly contributes to the development of atherosclerosis, but also activates MAPK, which increases the formation of free radicals, reduces the concentration of nitric oxide by increasing ET-1, and, in a vicious circle mechanism, increases insulin resistance [55]. The activity of the PI3-K/Akt pathway is also stopped, which, in the state of insulin resistance, is responsible for limiting the processes related to the development of atherosclerosis. The disturbed balance between PI3-K/Akt and MAPK leads to increased insulin resistance, endothelial cell dysfunction, and reduced nitric oxide production, and further to the development of obesity, diabetes, atherosclerosis, and an increased risk of cardiovascular diseases [56].

Obesity and insulin resistance, which are the hallmarks of type II diabetes, may lead to the inappropriate activation of macrophages and their accumulation of excess cholesterol, contributing to the promotion of the formation of atherosclerotic plaques. Additionally, through increased production of proteases, they contribute to increasing the instability of atherosclerotic lesions [57].

Increased levels and activity of dipeptidyl peptidase 4 (DPPP4) inhibitors have been demonstrated in patients with insulin resistance and type II diabetes. The conducted research further demonstrated that this enzyme participates in the regulation of lipid metabolism by increasing the deposition of ectopic fat particles in blood vessels; affects insulin levels, leading to reduced glucose consumption; and directly contributes to the induction of oxidative stress by affecting the dysfunction of the mitochondria and vascular endothelium. It also increases the level of free oxygen radicals. Due to all the functions involved in the promotion of atherosclerotic plaque, it seems to be the next breakthrough therapeutic target in the treatment of atherosclerosis [57].

#### 3.1.5. Cytokines—TGF-β Family

In recent years, published works have examined the impact of individual cytokines on the development of atherosclerotic plaques in more detail. It turned out that the previous views on the atheroprotective effect of the TGF-β and GDF-15 cytokine family were not entirely true. Agnė Liuizė Abramavičiūtė et al., in a published review, state that the levels of TGF-β1 were higher in a group of patients with atherosclerotic coronary artery disease compared to the control group. Subsequent studies confirmed a positive correlation between the level of TGF-β1 and higher blood pressure values, increased renin levels, and the accelerated aging of arteries, as well as greater left ventricular mass. TGF-β1 also negatively affects the activity of monocytes, contributing to the development of atherosclerotic plaques [58].

The influence of other cytokines from the TGF-β family—TGF-β2 and TGF-β3—is still poorly understood. It is suggested that high levels of TGF-β2 are associated with the greater stability of atherosclerotic plaque and a lower risk of future cardiovascular events. This cytokine appears to be a key point in the differentiation between stable and unstable atherosclerotic lesions. The effect of TGF-β3 is similarly poorly studied. Currently, it seems that examining the level of this cytokine in the blood may be important information for assessing the stability of plaques [59].

Another cytokine from the TGF-β family is growth differentiation factor 15 (GDF-15). In the human body, its expression is particularly visible in cardiomyocytes, macrophages, smooth muscle cells of the vascular wall, endothelial cells, and adipose tissue. Studies suggest that GDF-15 is involved in the initiation and progression of atherosclerosis. Studies conducted in vitro and in vivo have shown that the combination of GDF15 with oxidized lipid molecules increases the activity of macrophages, contributing to the progression of atherosclerosis. Liuizė Abramavičiūtė A et al., in a published review, cite a study in which the level of GDF-15 was compared in a group of patients with moderate-to-severe psoriasis with or without overlapping cardiovascular disease. It was shown that the group of patients without atherosclerotic disease had significantly lower levels of GFD than the study group [58,60].

#### 3.1.6. Copeptin

Copeptin is a protein released mainly in the hypothalamus, but also in the adrenal glands, testes, and sympathetic ganglia. In terms of structure, it is the C-terminal glycosylated part of provasopressin with a length of 39 amino acids and a molecular weight of 5 kDa with a core rich in lecin. It is secreted into the blood together with the other components of provasopressin—vasopressin and neurophysi II [61]. Copeptin secretion is regulated by the same factors as vasopressin secretion [62]. The strongest factor causing an increase in the secretion of both hormones is an increase in plasma osmolarity and a decrease in vascular volume [63]. It is involved in regulating the release of adrenocorticotropic hormone, regulating osmosis, and maintaining the homeostasis of the cardiovascular system [64]. Due to the limitations of measuring vasopressin concentration in blood, such as rapid degradation in vitro or pulsatile vasopressin secretion, copeptin level tests seem to be a good alternative [64,65,66,67,68]. Serum copeptin levels are less dependent on factors such as age and gender; they are characterized by low specificity (in many pathological conditions, its level may be increased) but high sensitivity [69]. Compared to vasopressin, it is more stable in serum and correlates better with plasma osmolarity than vasopressin [64].

Increased levels of copeptin can be seen in endocrine diseases such as diabetes insipidus and the syndrome of inappropriate secretion of vasopressin (SIADH) [64,70]. Copeptin also reflects the body’s stress response by influencing the hypothalamic–pituitary–adrenal axis, and therefore shows a positive correlation with blood cortisol concentration. In terms of the endocrine system, it may also play a role in the pathophysiology of diabetes—it affects insulin resistance and glucose homeostasis [71,72,73].

It may also be a prognostic marker in the event of acute stroke. The level of copeptin reflects the severity of the stroke, the probability of mortality, and the occurrence of complications after the disease. The higher the copeptin concentration, the greater the chance of a more severe course of the disease. A similar relationship was found in the case of intracerebral and subarachnoid hemorrhages. In all of these conditions, copeptin can be used as a marker [74].

Studies have also shown that copeptin levels are correlated with kidney failure. The level of GFR inversely reflects the concentration of copeptin—the reasons for this are, on the one hand, renal failure leading to a decrease in GFR and an increase in copeptin, and on the other hand, the increased concentration of AVP secreted in response to the overhydration of the body. A positive correlation was observed for copeptin and microalbuminuria, which is also one of the factors assessed in the course of renal failure [71,75,76]. Copeptin levels also appear to function as a biomarker in autosomal dominant polycystic kidney disease (ADPKD) [77].

Copeptin can also be used in perinatology. The correct levels of AVP and cortisol in newborns are necessary to maintain appropriate systemic pressure, body water management, proper surfactant synthesis, and homeostasis. Therefore, maintaining the above-mentioned hormones at the appropriate levels is very important because this protects against the stress reaction. Unfortunately, there are many clinical situations in which the levels of AVP, cortisol, and, as it recently turned out, copeptin, significantly exceed the upper limit of normal. These conditions include prolonged labor and vaginal delivery. Increased levels of AVP and copeptin may indicate perinatal ischemia and, consequently, hypoxia, which in turn leads to cerebral edema and increased intracranial pressure, which significantly worsens the prognosis. Due to the scientific evidence presented, copeptin is currently the most sensitive biomarker of labor stress [78].

Finally, copeptin may be used in a number of cardiovascular conditions. Studies have shown that copeptin levels assessed a few days before the onset of acute myocardial infarction were strongly correlated with left ventricular muscle dysfunction. Another study showed that copeptin levels were strongly correlated with 180-day mortality after an episode of acute myocardial infarction. Assessment of cardiac troponins will continue to be the gold standard for excluding myocardial infarction, but a strategy of assessing both troponins and copeptin appears to be more likely to effectively exclude acute myocardial infarction than measuring troponins alone [79,80,81].

Copeptin has also been proposed as a marker of heart failure. The conducted research showed that an increased level of copeptin correlates with a greater likelihood of developing heart failure. Interestingly, this relationship concerned heart failure with reduced systolic function. No similar phenomena were found for heart failure with preserved systolic function. Copeptin was found to be the strongest independent predictor of short-term (30-day) mortality in cohort studies. It can also be used to assess early complications of heart failure—dysfunction, volume, and left ventricular remodeling. Also in this case, it is believed that copeptin will not replace the currently used heart failure markers—NT-proBNP, hs-cTnT, and others—but measuring it together with other markers may improve diagnosis, assessment of complications, and patient stratification [80,81].

In the studies carried out, the level of copeptin was strongly correlated with the development of atherosclerosis and diabetic kidney disease in patients with diabetes. This relationship is particularly visible in patients with type II disease. Type I also appears to have some relationship but this has not yet been as widely confirmed [82]. Increased levels of copeptin lead to impaired glucose metabolism by intensifying the process of gluconeogenesis and glycogenolysis, as well as lipids—increasing the level of triglycerides and reducing the level of HDL cholesterol, these changes are closely correlated with the development of metabolic syndrome, abdominal obesity, and atherosclerosis [83]. Further, high levels of copeptin lead to albuminuria, reduced glomerular filtration in the kidneys, increased calcification in the coronary vessels, and increased levels of uric acid, which in turn is one of the indicators of hypertension. All of these individual conditions are associated with the development of atherosclerotic plaques [84].

A study by Martin Möckel and colleagues showed that the correlation of a negative copeptin result with a negative troponin result helps to better identify patients who may be eligible for outpatient treatment. The method of using two biomarkers to assess the prediction of the development of AMI is more detailed than using each of these markers individually; it shortens the patient’s hospital stay and increases the percentage of patients correctly discharged from the hospital. It should also be mentioned that a more detailed assessment of the risk of developing the disease using two markers is not characterized by an increased clinical danger for the patient compared to the determination of a single marker [85].

Similarly, a study conducted by Basak Karbek et al. showed that in a group of patients suffering from polycystic ovary syndrome (PCOS), the combination of cardiac troponin levels with copeptin levels has a greater diagnostic value in the development of acute myocardial infarction (AMI). Levels of both indicators below the accepted norm limit are characterized by a high negative predictive value of the development of AMI. The authors of the study also cite a work in which it was proven that copeptin has a better predictive value than cardiac troponin in relation to the development of stroke and AMI and related death [86].

#### 3.1.7. Homocysteine

Homocysteine is an amino acid produced in the human body as a result of methionine metabolism [87]. Physiologically, it occurs in blood at a concentration of 5–15 μmol/L. It is involved in two metabolic pathways: the methionine cycle and the trans-sulfuration sequence. These pathways are, respectively, responsible for the regeneration of homocysteine and the catabolic breakdown of its excess. An elevated homocysteine level is an amount exceeding 15 μmol/L. Hyperhomocystinemia may be associated with the development of epilepsy, intellectual disorders, age-related macular degeneration, severe vascular fibrosis, and atherosclerosis [88]. In recent studies, homocysteine levels have been positively correlated with various complications during pregnancy—recurrent miscarriages, placental abruption, preeclampsia, preterm birth, intrauterine growth restriction, and gestational diabetes [89].

The promotion of atherosclerotic plaque formation as a result of hyperhomocystinemia is multifactorial. Homocysteine, by reducing the availability of nitric oxide, may lead to the impaired functioning of the vascular endothelium. It leads to the production of endothelin-1, which is a substance that constricts blood vessels. It also takes part in the synthesis of pro-inflammatory cytokines (MCP-1, IL-8, NF-κB), contributing to the intensification of inflammation, which is a key point in the development of atherosclerosis. It further increases oxidative stress and causes disturbances in lipid metabolism, leading to a reduction in high-density lipoproteins (HDLs) [90]. Its influence on changes in the composition of blood vessels is also suggested. It leads to the proliferation of smooth muscle cells of the vascular wall, an increase in collagen synthesis and, consequently, a deterioration in the elasticity of blood vessels. By inducing changes in vascular composition, hyperhomocystinemia may lead to vascular stiffness. Its effect has also been shown to increase the activity of HMG Co A reductase, which leads to increased cholesterol synthesis, which in turn is a key factor in the development of atherosclerosis and other cardiovascular diseases [91].

Increased homocysteine levels have been correlated in scientific studies not only with an increased risk of developing atherosclerosis but also with other cardiovascular diseases—hypertension, myocardial infarction, or stroke. Its prothrombotic effect has also been demonstrated through the increased production of thromboxane A2 and a pro-aggregation effect on platelets [92,93].

Changes in homocysteine concentrations are strongly correlated with the levels of folic acid and vitamin B12 in the body. The negative correlation between vitamin B12, folic acid, and homocysteine may have significant clinical implications and can be used to prevent many disease states, including cardiovascular diseases. Studies have shown that people with deficiencies of both vitamins experience a significant increase in homocysteine levels [94]. From the above relationship, we can conclude that in people with vitamin B12 deficiency, supplementing it may result in lower homocysteine levels and, consequently, reduce the risk of developing atherosclerosis and other diseases associated with hyperhomocystinemia [95]. The authors of ref. Angelini A et al. [96] report that therapy consisting of the administration of folic acid and vitamin B12 leads to a reduction in the progression of cervical vasoconstriction, improved functioning of the vascular endothelium, reduced risk of cardiovascular disease by 15% after 24 months of therapy, and a reduction in homocysteine levels of more than 20% when therapy was continued for longer than 24 months, with albuminuria also reduced [96]. Table 1 presents a summary of the discussed biomarkers and their selected functions in the body.

### 3.2. Microelements and VitaminD

Herein, we describe the functions of iron, copper, zinc, and Vitamin D in the process of atherosclerotic plaque development.

#### 3.2.1. Iron

Iron is one of the microelements, extremely important in many metabolic pathways. In the body, it mainly occurs in the form of Fe^2+^ and Fe^3+^ ions. In the blood, Fe^3+^ ions are transferred to tissues using the transport protein transferrin. Within cells, they bind to the type 1 transferrin receptor, which allows them to enter the cell. Finally, after being converted into Fe^2+^ ions using STEAP3, they are stored using the ferritin protein. Disturbances in any stage of iron metabolism in the body lead to its excessive accumulation [102]. Too high a level of iron in the body can be very toxic to its functioning and lead to many diseases, including, as it turns out, atherosclerosis [103,104].

The mechanism by which free iron ions induce the formation of atherosclerotic plaques is the Fenton reaction, in which iron can cause the oxidation of various biological matrices in the body, resulting in the formation of free oxygen radicals, hydroxyl radicals, and iron ions in higher oxidation states. They react with LDL lipoprotein particles, causing their oxidation and further increased deposition within the blood vessel walls. Increased levels of iron in the body and oxidized LDL particles also contribute to their increased uptake by macrophages and, consequently, the faster formation of foam cells. Another mechanism is the intensification of the body’s inflammatory response by inducing oxidative stress. This process leads to damage to vascular endothelial cells, which, as we mentioned above, is one of the key capture points for circulating LDL particles [105,106]. Increased ferric ion concentration also causes the overexpression of individual inflammatory factors such as ICAM-1, VCAM-1, and MCP1, which further increase the overall inflammation [107].

Tasic et al., in their study, proved that patients with hemorrhagic atherosclerotic plaques had significantly higher levels of ferric ions in their serum compared to patients from the control group. The body’s iron metabolism, defined as the levels of transferrin and ferritin, is closely correlated with heart muscle diseases such as acute infarction. Animal studies have shown that reducing iron stores significantly reduces not only the risk of developing atherosclerotic lesions but also increases the stability of existing plaques and reduces their size. The above relationships were more strongly correlated in men than in women. The reasons for this state of affairs have not been fully determined; they are probably related to differences in the physiology of both sexes—hormone secretion or menstruation [108].

The concentrations of other metals in the body are extremely important because they work synergistically with free iron ions to form atherosclerotic plaques. The co-occurrence of an excess of Cu^2+^ ions with Fe^2+^ leads to the development of atherosclerotic plaques much faster than the mere presence of an increased concentration of ferric ions [106]. A similar relationship has been described for zinc ions [107]. It is worth mentioning that iron levels are also important not only for the development of atherosclerosis but also for other cardiovascular diseases such as acute heart attack, heart failure, cardiomyopathy, or hypertrophic hypertrophy of cardiac muscle cells [108].

A study by Ralph G. DePalma et al. showed that iron levels, defined by serum ferritin levels, have a direct relationship with inflammatory markers, interleukin 6, and CRP. Both ferritin and IL-6 levels were statistically higher in the group of patients who died. The correlation between ferritin and CRP has been confirmed in the general patient population and in the survivor population. Ferritin levels have also been shown to reflect the risk of death—the group of patients who died had significantly higher ferritin levels compared to those who survived [109].

#### 3.2.2. Copper

Copper is a microelement that is very important for many metabolic processes taking place in the human body. In the recent past, a new form of cell death dependent on copper ions was discovered—cuprostosis. Cuproptosis is cell death induced by the accumulation of copper ions in the mitochondria. This fact only confirmed the importance of copper metabolism for maintaining body homeostasis. Disturbances in the balance of copper concentration in the body can lead to diseases such as Wilson’s disease, Menkes’ disease, various liver diseases, and atherosclerosis [110].

The direct impact of copper ions on the development of atherosclerotic plaques is difficult to determine. Certainly, copper ions show some synergism with ferric ions as described above. Current research suggests that high levels of copper in the body induce mitochondrial changes that are responsible for endothelial cell dysfunction, oxidative stress, and an increased inflammatory response [110]. The increased level of intracellular copper ion concentration leads, through the activation of the NF-κB and AP-1 pathways, to the upregulation of CAMs and MCP-1 particles, which in turn increases the activity of endothelial cells [111]. Mohammad Ali Ghaffari et al. showed that copper ions can bind to LDL particles, increasing their susceptibility to oxidation. All these mechanisms may be important in the development of atherosclerotic plaques [111].

Elevated copper ion concentration has also been positively correlated with other cardiovascular diseases, especially coronary artery disease, ischemic heart disease, and acute myocardial infarction [112].

As it turns out, copper deficiency in the body may also contribute to the development of atherosclerotic plaques. Copper deficiency may be associated with the reduced antioxidant activity of cells. Studies on animal models confirm the above relationship. In studies conducted on humans, the link is not so obvious. Some research suggests an association between appropriate supplementation and a reduced risk of cardiovascular disease overall [112].

#### 3.2.3. Zinc

The presence of intracellular zinc is an important element regulating the activity of endothelial nitric oxide synthase (eNOS). eNOS is responsible for the synthesis of nitric oxide (NO) from arginine. Therefore, the increased release of zinc ions from eNOS causes a decrease in NO synthesis by limiting the activity of eNOS and, consequently, disturbances in the regulation of endothelial function, which in turn is one of the key elements in the development of atherosclerosis [113].

Zinc also has anti-inflammatory, anti-apoptotic, and antioxidant properties. Zinc ion deficiency leads to the induction of the apoptosis process. Its chronic deficiency leads to the suppression of c-Jun N-terminal kinase (JNK), which in turn is one of the factors of VSMC proliferation. Ensuring an appropriate amount of zinc by suppressing the activity of the NF-κB pathway is one of the elements that protect against the calcification process within VSMCs [113].

Studies have also shown that changes in zinc ion concentrations affect the expression of individual zinc ion transporters. Two transporters are of particular importance, Zip2 and Zip12, the expression of which increases significantly with zinc deficiency. Dysregulation within these two transporters has been positively correlated with the occurrence of cardiovascular diseases such as pulmonary hypertension and carotid artery disease. Zinc transporters are also involved in glucose and lipid metabolism. Dysfunction in zinc ion transporters leads to insulin resistance, which may further cause diabetes [114].

Similar relationships apply to zinc ions themselves—low levels have been associated with a greater risk of cardiovascular diseases, while high concentrations have shown a reduced risk of developing heart disease. It has been shown that maintaining an appropriate concentration is responsible for reducing the concentration of triglycerides, LDL particles, and cholesterol. Recent studies have proven that the long-term administration of small amounts of zinc, lasting over 12 weeks, has a positive effect on blood glucose levels, limiting the possibility of developing diabetes. All the above effects effectively limit the development of atherosclerosis and other cardiovascular diseases [114].

#### 3.2.4. Vitamin D

There are two main sources of vitamin D for the human body. The first one is supplied with food. The second is an endogenous source of the vitamin, which is produced from its precursor 7-dehydrocholesterol, which is found in skin cells under the influence of UVB radiation. Once it enters the body, it is metabolized in the liver by the enzyme 25-hydroxylase to 25-hydroxyvitamin D, also called calciferol (25(OH)D). 25(OH)D has a long half-life of 15 days. Then, this compound is hydroxylated in the kidneys by 1α-hydroxylase, creating the second active metabolite—1,25(OH)_2_D. Regulation of 1,25(OH)_2_D concentrations is possible using the 24-hydroxylase enzyme, which is responsible for the breakdown of the metabolite [115]. The basic function of vitamin D is to regulate the level of calcium in the blood and, consequently, the appropriate metabolism of bone tissue. Vitamin D receptors have been found in many other tissues, suggesting that it also has many extraskeletal actions [115].

Studies have shown that vitamin D deficiency may be correlated with the development of atherosclerotic plaques. Vitamin D modulates the body’s inflammatory response by reducing the synthesis of pro-inflammatory cytokines—IL-6, IL-1, IL-8, and TNF-alpha—and also reduces the expression of metalloproteinases 2 and 9, ensuring the greater stability of already formed plaques [116]. Inhibiting the induction of the inflammatory response, reducing the intensity of oxidative stress, limiting the expression of scavenger receptors, reducing autophagy generated by the PTPN6/SHP-1 pathway, and reducing the uptake of oxidized LDL particles by macrophages leads to a reduction in the formation of foam cells [116]. Vitamin D also participates in the regulation of NO synthesis dependent on nitric oxide synthetase [117].

Vitamin D metabolites also stimulate the formation of vascular endothelial growth factor and affect smooth muscle cells of the vascular wall, regulating their proliferation, migration, and expression of other tissue factors. The impaired signaling of vitamin D pathways was also associated with increased migration and interaction between leukocytes and vascular endothelial cells [117].

Observational studies have shown that vitamin D deficiency correlated with an unfavorable lipid profile. Vitamin D levels have been shown to be inversely correlated with total cholesterol, LDL, and triglycerides. Subsequent studies have proven similar relationships involving a reduction in triglyceride levels and an increase in HDL levels after vitamin D supplementation [118].

Despite the documented atheroprotective effect of vitamin D, the role of its supplementation in the treatment of atherosclerosis is still not clearly documented. In people without comorbidities, daily supplementation has not been shown to be associated with any changes in reducing cardiovascular risk. However, in the group of patients with other diseases, such as chronic kidney disease, type II diabetes, and coronary heart disease, it has been proven that supplementation of vitamin D or its active metabolites has a positive effect on the function of the vascular endothelium and alleviates inflammation. A meta-analysis conducted on 2000 patients with vitamin D deficiency showed an increased thickness of the carotid arteries and the incidence of atherosclerotic plaques compared to the control group [119].

Many studies have also correlated lower 25(OH)D concentrations with an increased risk of death from heart failure. Going further, it has been shown that its deficiencies are associated with shorter survival in this group of patients. Subsequent observational studies have linked deficiencies of active vitamin D metabolites with a greater risk of myocardial infarction and changes in left ventricular geometry leading to its hypertrophy [120]. Table 2 presents a summary of the discussed biomarkers and their selected functions in the body.

## 4. Conclusions

Atherosclerosis is a lifestyle disease that significantly increases cardiovascular risk and shortens survival time in various groups of patients. Detecting atherosclerosis at the subclinical stage can significantly reduce mortality by reducing the likelihood of cardiovascular events. The markers that we have proposed in the publication below as methods for early diagnosis of atherosclerosis have been proven in scientific research to influence the process of developing atherosclerotic plaques and maintaining their stability. Additionally, the exact normal ranges of the given biomarkers require standardization, both in the population at risk of atherosclerosis and in people who already suffer from it. Similarly, their clinical utility requires standardization compared to the currently available well-known predictors of atherosclerosis development. Modification of the metabolism of these compounds and greater focus on their impact on the development of atherosclerosis may in the future contribute to a breakthrough in the diagnosis and treatment of atherosclerotic plaques. Further research is needed on the impact of individual compounds and the potential possibility of their use to determine the development of atherosclerosis. Subsequent research should also largely focus on the early identification of people at risk of developing atherosclerosis and, consequently, the quick identification of possible factors inducing the development of plaque. For example, it may turn out that a high level of iron in the serum is responsible for the early stages of plaque formation, and an appropriate quick identification of this group of diseases and early use of drugs lowering the level of iron in the serum will prove to be the fastest, safest, and cheapest preventive method. It should be remembered that the current standard, which is the chronic use of statins to long-term lower blood cholesterol levels, is not a treatment without side effects. Statins significantly damage the muscles and liver. Limiting their use at the expense of preventive use of other groups of drugs may be necessary in groups of patients who show undesirable effects after using statins and in people who are not eligible for invasive procedures. It is also necessary to search for additional markers that are currently not taken into account in the context of atherosclerosis. Atherosclerosis is a systemic disease, it can affect any artery in the body, as has already been proven in research, its etiopathogenetic basis is multifactorial and complicated; for these reasons, the approach to the diagnosis and treatment of atherosclerosis should also be multifactorial and take into account various reactions occurring in the body, which currently are not taken into account.

## Figures and Tables

**Table 1:** Early markers for the diagnosis of atherosclerosis- CRP, Osteopontin, Osteoprotegerin, Copeptin and Homocysteine.

Biomarker	Structure	Source	Selected Functions in the Body	Pathomechanism	Selected Other Disease States Associated with Increased/Decreased Biomarker Levels (Other than Cardiovascular Diseases)
CRP	Protein	Synthesized in the liver in response to signals sent by inflammatory cells.	Inflammatory biomarker and one of the mediators of the acute phase reaction [97].	Intensification of inflammation by increasing the production of pro-inflammatory cytokines and reducing NO production.	Autoimmune diseases, cancer, acute infectious diseases, liver cirrhosis, generalized inflammatory response syndrome [97].
Osteopontin	Protein	It is produced in the human body as a response to hypocalcemia and hypophosphatemia in the blood. It occurs in many different cells.	It is involved in the inflammatory response, wound healing, maintaining proper metabolic homeostasis of the cartilage and subchondral layer, and regulating the metabolism of chondrocytes and osteocytes [98].	Reduces the availability of NO. Induces inflammation. Induces the proliferation of VSMCs. It causes an increased influx of inflammatory cells into the forming plaque.	Diabetes and prediabetes, obesity, chronic kidney disease, liver diseases, subarachnoid bleeding, rheumatological diseases [98].
Osteoprotegerin	Protein	Produced in the human body by cardiomyocytes, epithelial cells, lungs, bones, immune system cells, and kidneys.	It takes part in the metabolism of bone tissue. It regulates the process of proliferation and differentiation of osteoclasts. It exerts an inhibitory effect by blocking the binding of particles to RANKL [99].	Calcification of vessels. Promotion of adhesion of leukocytes and other inflammatory cells to the vascular wall. Increased activity of metalloproteinases leading to plaque instability.	RA, Crohn’s disease, Kawasaki disease, lupus, type 2 diabetes and related insulin resistance, metabolic syndrome, NAFLD.
Copeptin	Peptide	Hypothalamus adrenal glands, testes, sympathetic ganglia.	Regulation of ACTH release, osmosis process, and maintaining homeostasis of the cardiovascular system [70].	It impairs lipid metabolism, leading to increased triglyceride levels and decreased HDL levels.	Endocrine diseases—SIADH, diabetes. Stroke, renal failure, ADPKD, perinatal ischemia, increased intracranial pressure.
Homocysteine	Amino acid	It is produced in the human body as a result of methionine metabolism.	It takes part in cellular metabolism and protein synthesis.	Reducing the availability of NO. Constriction of blood vessels. Synthesis of pro-inflammatory cytokines. Intensification of oxidative stress. VSMC proliferation.	Gynecological diseases—miscarriages, placental abruption, intrauterine growth restriction, gestational diabetes. Neurological diseases—Parkinson’s disease, Alzheimer’s disease, epileptic disorders. Psoriasis, kidney disease [100,101]

**Table 2:** Early markers for the diagnosis of atherosclerosis- Iron ion, Copper ion, Zinc ion, Vitamin D.

Biomarker	Structure	Source	Selected Functions in the Body	Pathomechanism	Selected Other Disease States Associated with Increased/Decreased Biomarker Levels (Other than Cardiovascular Diseases)
Iron ion	Ion	External sources.	It is a component of hemoglobin, myoglobin, and electron transport chain proteins. It is a catalyst for many enzymatic reactions [121].	Free ferric ions lead to the induction of oxidative stress by increasing the amount of ROS and LDL oxidation.	Non-alcoholic steatohepatitis, Parkinson’s disease, Alzheimer’s disease, colon cancer, breast cancer [122].
Copper ion	Ion	External sources.	It is a cofactor of many enzymes, mainly oxygenases, oxidoreductases, transferases, and hydroxylases [123].	Synergistic effect with ferric ions. Induction of mitochondrial changes.	Hemolytic anemia, PCOS, ovarian cancer, cervical cancer, endometrial cancer [124]
Zinc ion	Ion	External sources.	It has anti-inflammatory, anti-apoptotic, and antioxidant properties and is an important element in neurotransmission and neurogenesis. It takes part in the regulation of the cell cycle and the process of apoptosis [125].	Induction of apoptosis. Proliferation of VSMCs.	HIV infection, chronic kidney disease, liver disease, PCOS, thalassemia [126].
Vitamin D	Vitamin	External sources. Also produced in the human body in the skin under the influence of solar radiation from its precursor 7-dehydrocholesterol.	It reduces PTH secretion, increases calcium absorption, supports osteoblast function and osteoclastic bone resorption. It has a number of non-skeletal effects—it increases insulin levels, supports the immune system, strengthens muscle function, and supports the process of cell differentiation [127].	External sources. Also produced in the human body in the skin under the influence of solar radiation from its precursor 7-dehydrocholesterol.	Arterial hypertension, diabetes and prediabetes, obesity, lipid metabolism disorders, neurological diseases—Alzheimer’s disease, Parkinson’s disease, amyotrophic lateral sclerosis, multiple sclerosis, migraine, schizophrenia, endocrine diseases [128].

## Data Availability

All data are included in the manuscript.
